# *Haemophilus influenzae* type b vaccination and anthropometric, cognitive, and schooling outcomes among Indian children

**DOI:** 10.1111/nyas.14127

**Published:** 2019-06-10

**Authors:** Arindam Nandi, Anil B. Deolalikar, David E. Bloom, Ramanan Laxminarayan

**Affiliations:** ^1^ Center for Disease Dynamics Economics and Policy Washington DC; ^2^ School of Public Policy and Department of Economics University of California Riverside California; ^3^ Department of Global Health and Population Harvard T.H. Chan School of Public Health Boston Massachusetts; ^4^ Center for Disease Dynamics Economics and Policy New Delhi India; ^5^ Princeton Environmental Institute Princeton University Princeton New Jersey

**Keywords:** India, *Haemophilus influenzae*, Hib vaccine, child development, young lives

## Abstract

*Haemophilus influenzae* type b (Hib) affects 337,000 Indian children every year. A vaccine against Hib was introduced in 2011 as part of the pentavalent vaccine and scaled up nationwide. This study investigated the associations between Hib vaccination and child anthropometry, cognition, and schooling outcomes in India. We used longitudinal survey data and employed propensity score matching to control for observed systematic differences between children who reported receipt or nonreceipt of Hib vaccine before age 6 years (*n* = 1824). Z‐scores of height‐for‐age (HAZ) and BMI‐for‐age (BMIZ), percentage scores of English, mathematics, reading, and Peabody Picture Vocabulary tests, and attained schooling grade of children were examined. Hib‐vaccinated children had 0.25 higher HAZ, scored 4.09 percentage points (pp) higher on the English test and 4.78 pp higher on the mathematics test, and attained 0.16 more schooling grades than Hib‐unvaccinated children at age 11–12 years. At age 14–15 years, they had 0.18 higher HAZ, scored 3.63 pp higher on the reading test and 3.22 pp higher on the mathematics test, and attained 0.15 more schooling grades than Hib‐unvaccinated children. The findings indicate potential long‐term health, cognitive, and schooling benefits of the Hib vaccine, subject to the effect of unobserved confounding factors.

## Introduction

*Haemophilus influenzae* type b (Hib) is a bacterial pathogen that causes pneumonia and bacterial meningitis and, to a lesser extent, epiglottitis and other infections among children under age 5.^1^, ^2^ Although the national burden of Hib has declined by 84% since 2000, India continues to have the largest number of Hib cases and associated deaths in the world.^3^ An estimated 337,270 Hib cases and 15,634 deaths occurred among Indian children aged 1–59 months in 2015;^3^ pneumonia and meningitis comprised 99% of the cases. The Hib vaccine has been privately available in India since 1997. In 2011, the Indian government introduced Hib immunization as part of the pentavalent vaccine, in combination with vaccines for diphtheria, tetanus, pertussis, and hepatitis B. The pentavalent vaccine was introduced on a trial basis in two states and currently covers 88% of children under the age of 2 years nationwide.^4^, ^5^

Infectious diseases in the first 5 years of life are frequently linked to worse health and nonhealth outcomes over the life cycle.^6^, ^7^ Childhood pneumonia—a small proportion of which is currently due to Hib—may increase the risk of reduced physical growth or stunting, diminished learning capacity, and lower economic productivity.^6^, ^7^, ^8^ It may also increase the adult risk of asthma, chronic obstructive pulmonary disease, and bronchiectasis.^9^, ^10^, ^11^ Episodes of Hib pneumonia in childhood—the most common form of Hib infection—may be associated with preschool or school absenteeism, thereby adversely affecting learning and schooling outcomes.^12^, ^13^, ^14^ Finally, bacterial meningitis in childhood, such as that caused by Hib, could impede brain development and cause neurological disabilities, deafness, blindness, and paralysis, although such severe consequences may be rare.^15^, ^16^, ^17^, ^18^, ^19^, ^20^, ^21^, ^22^, ^23^, ^24^

Most studies focus on the long‐term negative effects of Hib infection in high‐income countries, but these findings may not be generalizable to low‐ and middle‐income countries (LMICs), where the underlying socioeconomic conditions and disease burdens are different. To the best of our knowledge, only one study has used longitudinal data to investigate the question: it found that out of 1824 Indian children, those vaccinated against Hib were 24% less likely to be stunted and 21% less likely to be underweight at ages 4–6 years.^25^ The authors did not examine the nutritional status of children beyond this age, or their cognitive development or schooling outcomes at any age. The study also did not account for possible confounding effects from other vaccines.

In this study, we used longitudinal data from India and employed a quasiexperimental methodology that enabled us to address some of the shortcomings of the existing literature. We investigated the associations of Hib vaccination in early childhood with anthropometric, cognitive functioning, and schooling attainment outcomes for children at ages 11–12 and 14–15 years.

## Materials and methods

### Young lives survey data

We used data from the Young Lives Survey (YLS), an international study of the determinants and consequences of childhood poverty.^26^ In 2002, the study enrolled a “younger cohort” of approximately 2000 children aged 6–18 months and an “older cohort” of approximately 1000 children aged 7.5–8.5 years in four countries—Ethiopia, India, Peru, and Vietnam—for a total enrollment of approximately 12,000 children. The children and their households were resurveyed in 2006, 2009, 2013, and 2016.

We analyzed the younger‐cohort data from India in this study. In India, six districts from three agro‐climatic regions in the formerly undivided state of Andhra Pradesh (presently divided into Andhra Pradesh and Telangana) were selected for YLS.^27^ Twenty sentinel sites (subdistrict) were selected, and in each site, 100 households with one child in the younger cohort were chosen for the survey. At the 2002 baseline, 2011 children and their households were enrolled. A comparison based on the National Family Health Survey of India (1998–1999) shows that the YLS households were slightly wealthier and had better access to services as compared with the average household in these states, although secular trends may explain such differences.^28^, ^29^ Sample attrition between the baseline and the 2016 follow‐up surveys was 3%.

In 2006, when the younger cohort was 4–6 years old, the survey collected data on whether a child had received Hib vaccination within the past 4 years (as reported by the mother or primary caregiver). Vaccination card was reported to be available for 47% of children. Considering that the Hib vaccine was not included in the national child immunization program at that time, whether the card listed the vaccine is unknown. The survey also did not collect data on the whether the vaccine receipt information was cross‐validated with the card.

We combined the vaccine receipt information with household socioeconomic characteristics and child anthropometric, cognition, and schooling data from the 2013 and 2016 surveys. Community‐level information on access to public healthcare, such as the availability of health centers (which could affect vaccination rates), was available for 88% of children in 2002. We used these data for additional analyses, as discussed later.

Using the 2013 survey data, we examined the following anthropometric indicators of 11‐ to 12‐year‐old children: height‐for‐age Z‐scores (HAZ) and body‐mass‐index‐for‐age Z‐scores (BMIZ). We excluded children with outlier values of the indicators (HAZ < –6 or HAZ > 6 and BMIZ < –5 or BMIZ > 5).

We evaluated cognition indicators, which were based on percentage scores, from the following standardized assessments: an English language test, the Peabody Picture Vocabulary Test (PPVT), a local language test, and a mathematics test.^30^, ^31^ The English and local language tests involved answering questions based on words and passages. The mathematics test consisted of number recognition and basic operations, and the PPVT included a set of age‐appropriate questions that were adapted for India. The psychometric validity of these tests has been discussed in a previous YLS study.^30^ We also examined a schooling indicator: highest grade attained by the child, which ranged from 0 to 11 in the 2013 data.

Using the 2016 survey data—when the children were 14–15 years old—we examined the same indicators, with the exception of the English and local language test scores, which were not administered. Instead, we included the percentage score of a reading test, which was available in the 2016 data. In this survey round, the highest grade attained ranged from 1 to 12.

### Analysis

Childhood vaccination rates vary widely across regions and socioeconomic groups in India.^32^, ^33^, ^34^ Standard of living, access to postnatal care and subsidized vaccines, maternal schooling, and health insurance coverage have all been linked positively to vaccination rates.^35^, ^36^ These factors could make Hib‐vaccinated and ‐unvaccinated children systematically different, potentially biasing least squares regression–based estimates of the associations of the vaccine with our outcomes of interest because such differences may also be linked to these outcomes.

We used a quasiexperimental methodology of propensity score matching (PSM) to reduce the differences in the characteristics of the two groups of children.^37^, ^38^, ^39^, ^40^ Separately for the 2013 and 2016 survey rounds, we first regressed the Hib‐vaccinated status of each child on the following characteristics: age of child in months, squared value of age in months, whether child is female, whether child was born prematurely, rural household indicator, number of household members, and indicators of caste groups (scheduled caste, scheduled tribe, or other backward classes) and religion (Muslim, Christian, or Buddhist). We also included covariates for the characteristics of the household head: whether female, age in years, and indicators of schooling level (<6 years, ≥6 years but ≤11 years, and ≥12 years of schooling). Mother's age in years and schooling indicators (same categories as listed) are also included.

We measured the household standard of living through a composite YLS index of durable assets, such as television, radio, and bicycle ownership, along with housing conditions, such as indicators of construction quality and access to drinking water and toilets.^41^ We divided the wealth index into quintiles and included binary indicators of second to fifth quintiles in the regression.

Using the predicted probability (propensity score) of Hib vaccination from this probit model, we matched Hib‐vaccinated children with similar Hib‐unvaccinated children. We used a kernel (Epanechnikov) matching algorithm and only considered the observations with overlapping propensity scores (known as the “common support”). After matching, differences in child outcomes between the Hib‐vaccinated and matched Hib‐unvaccinated groups could be attributed to the Hib vaccine. This difference is known as the *average treatment effect on treated* (ATT).

In additional analyses, we estimated the ATT associations of the Hib vaccine by including the following public healthcare indicators in the probit model of propensity scores: availability of a public hospital, a public health post, and a public health center. We considered *P* < 0.05 for statistical significance in all models.

### Comparison with other vaccines, matching quality tests, and additional matching algorithms

We examined the associations of Hib vaccination vis‐à‐vis two other vaccines: the 2002 YLS collected information on the administration of bacillus Calmette–Guérin (BCG) and measles vaccines to the child (reported by the mother and cross‐validated with vaccination card). We estimated the ATT associations of Hib vaccine only within the subsample of children who had received BCG vaccine (92%). We repeated a similar analysis among measles‐vaccinated children (73%).

We also used multiple tests to check whether the kernel PSM algorithm was valid, that is, if it reduced the initial differences between the Hib‐vaccinated and ‐unvaccinated groups. First, we calculated the mean standardized percentage bias, that is, the average percentage difference in the values of PSM probit model covariates between Hib‐vaccinated and ‐unvaccinated groups before matching. We then compared it with the mean standardized bias calculated from the Hib‐vaccinated and matched Hib‐unvaccinated (after matching) groups. A valid PSM would reduce the mean bias substantially pre‐ to post‐matching.

Second, we reestimated the PSM probit model using the Hib‐vaccinated and matched Hib‐unvaccinated samples. If PSM successfully reduced the differences between the two groups, the pseudo‐*R* 2 statistic from this regression should be substantially smaller than the pseudo‐*R* 2 in the original model.

Third, we conducted a likelihood ratio test of the joint significance of the probit covariates before and after matching. In the matched Hib‐vaccinated and ‐unvaccinated sample, failure to reject the null hypothesis of joint significance would indicate that the quality of matching was high, that is, the covariates could no longer explain the Hib‐vaccination status of a child in the combined matched data.

Finally, we tested the sensitivity of our results by repeating the analysis under two other matching algorithms: one‐to‐one nearest neighbor matching with replacement and within a probability radius of 0.01 and one‐to‐three nearest neighbors matching.

## Results

Our sample included 1824 younger‐cohort children, of whom 42.9% were reported as Hib‐vaccinated. Table 1 presents the summary statistics of the study sample by Hib vaccination status. As compared with Hib‐unvaccinated children, Hib‐vaccinated children were more likely to be younger, female, from smaller and wealthier families, from urban areas, and have younger mothers and household heads.

**Table 1:** Summary statistics of YLS younger cohort in India

	2013 Survey (11–12 year olds)		2016 Survey (14–15 year olds)	
	Hib‐vaccinated	Hib‐unvaccinated	Hib‐vaccinated	Hib‐unvaccinated
	Mean ± SD	Mean ± SD	Mean ± SD	Mean ± SD
Height‐for‐age Z‐score (HAZ)	−1.3 ± 1	−1.6 ± 1	−1.3 ± 0.9	−1.6 ± 1
BMI‐for‐age Z‐score (BMIZ)	−1.3 ± 1.4	−1.4 ± 1.3	−1 ± 1.4	−1.2 ± 1.3
English percentage score	66 ± 18.5	58.6 ± 20.5	–	–
PPVT percentage score (0–100)	76 ± 13.5	75.3 ± 14	83.8 ± 14.3	82.7 ± 13.5
Language percentage score (0–100)	57.8 ± 18.2	54.7 ± 18.8	–	–
Reading percentage score (0–100)	–	–	58.3 ± 17.2	52.9 ± 17.3
Mathematics percentage score (0–100)	47.8 ± 22.4	41.5 ± 22.8	36.1 ± 17.1	31.2 ± 15.9
Highest schooling grade attained	5.5 ± 1.2	5.4 ± 1.4	8.5 ± 1.2	8.3 ± 1.3
Age of child in months	143.7 ± 3.8	143.9 ± 3.8	179.8 ± 3.8	180.1 ± 3.8
Whether child was born premature	0.1 ± 0.3	0.1 ± 0.3	0.1 ± 0.3	0.1 ± 0.3
Whether child is female	0.5 ± 0.5	0.4 ± 0.5	0.5 ± 0.5	0.4 ± 0.5
Rural household	0.6 ± 0.5	0.8 ± 0.4	0.6 ± 0.5	0.8 ± 0.4
Household size	4.8 ± 1.5	4.9 ± 1.9	4.6 ± 1.5	4.9 ± 1.8
Whether scheduled caste (SC)	0.2 ± 0.4	0.2 ± 0.4	0.2 ± 0.4	0.2 ± 0.4
Whether scheduled tribe (ST)	0.2 ± 0.4	0.1 ± 0.3	0.2 ± 0.4	0.1 ± 0.3
Whether other backward classes (OBCs)	0.5 ± 0.5	0.5 ± 0.5	0.5 ± 0.5	0.5 ± 0.5
Whether Muslim	0.1 ± 0.3	0.1 ± 0.2	0.1 ± 0.3	0.1 ± 0.2
Whether Christian	0 ± 0.2	0.1 ± 0.2	0 ± 0.2	0.1 ± 0.2
Whether Buddhist	0 ± 0.1	0 ± 0.1	0 ± 0.1	0 ± 0.1
Whether household head is female	0.1 ± 0.3	0.1 ± 0.3	0.2 ± 0.4	0.1 ± 0.3
Age of household head in years	41.1 ± 7.7	41.4 ± 8.1	43.2 ± 7.3	43.6 ± 7.8
Head's schooling: literate but <6 years	0.2 ± 0.4	0.2 ± 0.4	0.3 ± 0.4	0.3 ± 0.5
Head's schooling: ≥6 years but ≤11 years	0.3 ± 0.5	0.3 ± 0.5	0.4 ± 0.5	0.4 ± 0.5
Head's schooling: ≥12 years	0.4 ± 0.5	0.3 ± 0.4	0.2 ± 0.4	0.1 ± 0.4
Mother's schooling: literate but <6 years	0.1 ± 0.4	0.2 ± 0.4	0.2 ± 0.4	0.2 ± 0.4
Mother's schooling: ≥6 years but ≤11 years	0.3 ± 0.5	0.2 ± 0.4	0.3 ± 0.5	0.2 ± 0.4
Mother's schooling: ≥12 years	0.2 ± 0.4	0.2 ± 0.4	0.1 ± 0.4	0.1 ± 0.3
Mother's age in years	34.3 ± 4	34.8 ± 4.5	37.3 ± 4.1	37.8 ± 4.5
Household belongs to wealth quintile 2	0.2 ± 0.4	0.3 ± 0.4	0.2 ± 0.4	0.2 ± 0.4
Household belongs to wealth quintile 3	0.2 ± 0.4	0.2 ± 0.4	0.2 ± 0.4	0.2 ± 0.4
Household belongs to wealth quintile 4	0.2 ± 0.4	0.2 ± 0.4	0.2 ± 0.4	0.2 ± 0.4
Household belongs to wealth quintile 5	0.3 ± 0.4	0.1 ± 0.3	0.3 ± 0.4	0.1 ± 0.3
Sample size	783	1041	783	1041

note: Data are from 2013 and 2016 rounds of the Young Lives Survey, comprising 1824 children in the younger cohort in India. The sample is divided based on Hib vaccination information from the 2006 round. SD denotes standard deviation. Empty cells indicate outcome variables not measured in that survey round.

### Outcomes at ages 11–12 years (2013 YLS)

Table 2 presents estimates of the ATT associations of Hib vaccination. When public healthcare access indicators were excluded from propensity score estimation (Model 1), Hib‐vaccinated children had 0.25 (95% CI: 0.14–0.35; *P* < 0.001) higher HAZ, scored 4.09 (95% CI: 2.02–6.15; *P* < 0.001) percentage points (pp) higher on the English test and 4.78 pp (95% CI: 2.41–7.15; *P* < 0.001) higher on the mathematics test, and attained 0.16 (95% CI: 0.03–0.3; *P* < 0.05) more schooling grades than matched Hib‐unvaccinated children.

**Table 2:** Propensity score matching–based estimates (ATT) of the associations of Hib vaccination

	Model (1)		Model (2)	
	ATT	*P* value	ATT	*P* value
2013 Survey (11–12 year olds):				
Height‐for‐age Z‐score (HAZ)	0.25	0.00	0.22	0.00
BMI‐for‐age Z‐score (BMIZ)	−0.05	0.48	−0.03	0.71
English percentage score (0–100)	4.09	0.00	4.01	0.00
PPVT percentage score (0–100)	−0.56	0.44	−0.52	0.56
Language percentage score (0–100)	1.63	0.10	−0.01	0.99
Mathematics percentage score (0–100)	4.78	0.00	3.70	0.01
Highest schooling grade attained	0.16	0.02	0.14	0.11
2016 survey (14–15 year olds):				
Height‐for‐age Z‐score (HAZ)	0.18	0.00	0.17	0.01
BMI‐for‐age Z‐score (BMIZ)	0.07	0.35	0.11	0.20
PPVT percentage score (0–100)	−0.21	0.77	0.14	0.87
Reading percentage score (0–100)	3.63	0.00	2.67	0.02
Mathematics percentage score (0–100)	3.22	0.00	2.76	0.01
Highest schooling grade attained	0.15	0.02	0.15	0.07

note: Data are from 2013 and 2016 rounds of the Young Lives Survey, comprising 1824 children in the younger cohort in India. ATT denotes the propensity score matching estimator (kernel) of the associations of Hib vaccination. Model (1) excluded public healthcare access indicators from propensity score estimation, while Model (2) included them.

After including public healthcare access indicators, which were available for 88% of the sample (Model 2), Hib‐vaccinated children had 0.22 (95% CI: 0.09–0.34; *P* < 0.01) higher HAZ and scored 4.01 pp (95% CI: 1.48–6.53; *P* < 0.01) higher on the English test and 3.7 pp (95% CI: 0.77–6.62; *P* < 0.05) higher on the mathematics test than matched Hib‐unvaccinated children.

### Outcomes at ages 14–15 years (2016 YLS)

In Model (1), Hib‐vaccinated children had 0.18 (95% CI: 0.08–0.28; *P* < 0.001) higher HAZ, scored 3.63 pp (95% CI: 1.81–5.44; *P* < 0.001) higher on the reading test and 3.22 pp (95% CI: 1.49–4.96; *P* < 0.001) higher on the mathematics test, and attained 0.15 (95% CI: 0.02–0.28; *P* < 0.05) more schooling grades than matched Hib‐unvaccinated children.

In Model (2), Hib‐vaccinated children had 0.17 (95% CI: 0.05–0.3; *P* < 0.01) higher HAZ and scored 2.67 pp (95% CI: 0.45–4.89; *P* < 0.05) higher on the reading test and 2.76 pp (95% CI: 0.65–4.87; *P* < 0.05) higher on the mathematics test than matched Hib‐unvaccinated children.

### Results from comparison with BCG and measles vaccines

Table 3 presents results from the BCG‐only and measles‐only analyses. Among BCG‐vaccinated 11‐ to 12‐year‐old children in Model (1), children who also received the Hib vaccine had 0.21 (95% CI: 0.1–0.31; *P* < 0.001) higher HAZ and scored 3.49 pp (95% CI: 1.51–5.46; *P* < 0.001) higher on the English test and 4.02 pp (95% CI: 1.73–6.32; *P* < 0.001) higher on the mathematics test than matched children who had received the BCG vaccine but not Hib. At age 14–15 years, these Hib‐vaccinated children had 0.17 (95% CI: 0.07–0.27; *P* < 0.001) higher HAZ and scored 3.52 pp (95% CI: 1.76–5.28; *P* < 0.001) and 3.15 pp (95% CI: 1.44–4.87; *P* < 0.001) higher on the reading and mathematics tests, respectively, than their matched counterparts.

**Table 3:** Comparison with BCG and measles vaccines

	BCG‐vaccinated children				Measles‐vaccinated children			
	Model (1)		Model (2)		Model (1)		Model (2)	
	ATT	*P* value	ATT	*P* value	ATT	*P* value	ATT	*P* value
2013 survey (11–12 year olds):								
Height‐for‐age Z‐score (HAZ)	0.21	0.00	0.18	0.00	0.15	0.01	0.15	0.03
BMI‐for‐age Z‐score (BMIZ)	−0.04	0.58	−0.01	0.88	−0.04	0.62	−0.02	0.79
English percentage score (0–100)	3.49	0.00	3.35	0.01	4.05	0.00	4.13	0.00
PPVT percentage score (0–100)	−0.61	0.39	−0.78	0.36	−0.34	0.66	−0.01	0.99
Language percentage score (0–100)	1.69	0.08	0.93	0.42	1.66	0.13	1.07	0.40
Mathematics percentage score (0–100)	4.02	0.00	3.30	0.02	3.42	0.01	2.38	0.13
Highest schooling grade attained	0.12	0.07	0.10	0.20	0.06	0.44	0.10	0.28
2016 survey (14–15 year olds):								
Height‐for‐age Z‐score (HAZ)	0.17	0.00	0.16	0.01	0.12	0.04	0.12	0.08
BMI‐for‐age Z‐score (BMIZ)	0.09	0.22	0.11	0.19	0.10	0.23	0.10	0.27
PPVT percentage score (0–100)	−0.20	0.78	−0.12	0.90	−0.85	0.30	−0.69	0.47
Reading percentage score (0–100)	3.52	0.00	2.69	0.01	3.84	0.00	3.05	0.01
Mathematics percentage score (0–100)	3.15	0.00	2.54	0.01	2.95	0.00	2.12	0.07
Highest schooling grade attained	0.12	0.06	0.12	0.12	0.10	0.17	0.13	0.13

note: Data are from 2013 and 2016 rounds of the Young Lives Survey's younger cohort in India. Only children who were reported as BCG‐vaccinated or measles‐vaccinated in 2002 were included. ATT denotes the propensity score matching estimator (kernel) of the associations of Hib vaccination. Model (1) excluded public healthcare access indicators from propensity score estimation, while Model (2) included them.

Among measles‐vaccinated children of age 11–12 years in Model (1), those who were also vaccinated with Hib had 0.15 (95% CI: 0.03–0.26; *P* < 0.05) higher HAZ and scored 4.05 pp (95% CI: 1.8–6.3; *P* < 0.001) and 3.42 pp (95% CI: 0.79–6.04; *P* < 0.05) higher on the English and mathematics tests, respectively, than matched children who had received the measles vaccine but not Hib. At age 14–15 years, these Hib‐vaccinated children had 0.12 (95% CI: 0–0.23; *P* < 0.05) higher HAZ and scored 3.84 pp (95% CI: 1.86–5.82; *P* < 0.001) and 2.95 pp (95% CI: 1–4.9; *P* < 0.01) higher on the reading and mathematics tests, respectively, than matched Hib‐unvaccinated children.

Results from Model (2) were similar to Model (1) for both the BCG‐only and measles‐only analyses. They are presented in Table 3 but not discussed here separately.

### Results from matching quality tests and alternative matching algorithms

Tables 4 and 5 present results from the tests of matching quality under the original kernel PSM Model (1) and Model (2), respectively. The mean standardized percentage bias declined by more than 74%, while pseudo‐*R* 2 declined by more than 95% from pre‐ to post‐matching in Model (1). Similarly, mean bias and pseudo‐*R* 2 declined by at least 73% and 93%, respectively, after matching in Model (2). In all models, the *P* value of the likelihood ratio *χ* ^2^ was higher than 0.05 after matching. This implies that the null hypothesis of joint significance of covariates in the propensity score estimation using the matched sample could not be rejected. Additional results presented in Tables 6 and 7 show that the standardized percentage bias reduced substantially from pre‐ to post‐matching for almost all individual covariates of the models.

**Table 4:** Model (1)—tests of matching quality

	Mean % bias in unmatched data	Mean % bias after PSM	Pseudo‐*R* ^2^ before matching	Pseudo‐*R* ^2^ after matching	*P* value for *χ* ^2^ before matching	*P* value for *χ* ^2^ after matching
Analysis of 2013 survey (11–12 year olds):						
Height‐for‐age Z‐score (HAZ)	11.6	2.6	0.079	0.003	0	1
BMI‐for‐age Z‐score (BMIZ)	11.6	2.6	0.079	0.003	0	1
English percentage score (0–100)	11.6	2.5	0.079	0.003	0	1
PPVT percentage score (0–100)	11.6	2.5	0.079	0.003	0	1
Language percentage score (0–100)	11.6	2.5	0.079	0.003	0	1
Mathematics percentage score (0–100)	11.6	2.5	0.079	0.003	0	1
Highest schooling grade attained	11.6	2.6	0.079	0.003	0	1
Analysis of 2016 survey (14–15 year olds):						
Height‐for‐age Z‐score (HAZ)	11.5	2.9	0.072	0.003	0	1
BMI‐for‐age Z‐score (BMIZ)	11.5	2.8	0.072	0.003	0	1
PPVT percentage score (0–100)	11.5	2.9	0.072	0.003	0	1
Reading percentage score (0–100)	11.5	2.8	0.072	0.003	0	1
Mathematics percentage score (0–100)	11.5	2.8	0.072	0.003	0	1
Highest schooling grade attained	11.5	3	0.072	0.003	0	1

note: Bias is measured as the standardized percentage difference in the average value of the variable between the Hib‐vaccinated and ‐unvaccinated samples. Matching was based on propensity scores, using the kernel (Epanechnikov) matching method. Model (1) excluded public healthcare access indicators from propensity score estimation.

**Table 5:** Model (2)—tests of matching quality

	Mean % bias in unmatched data	Mean % bias after PSM	Pseudo‐*R* ^2^ before matching	Pseudo‐*R* ^2^ after matching	*P* value for *χ* ^2^ before matching	*P* value for *χ* ^2^ after matching
Analysis of 2013 survey (11–12 year olds):						
Height‐for‐age Z‐score (HAZ)	19.9	4.2	0.171	0.008	0	0.986
BMI‐for‐age Z‐score (BMIZ)	19.9	4.2	0.171	0.008	0	0.987
English percentage score (0–100)	19.9	4.3	0.171	0.008	0	0.985
PPVT percentage score (0–100)	19.9	4.2	0.171	0.008	0	0.986
Language percentage score (0–100)	19.9	4.3	0.171	0.008	0	0.981
Mathematics percentage score (0–100)	19.9	4.3	0.171	0.008	0	0.983
Highest schooling grade attained	19.9	4.2	0.171	0.008	0	0.986
Analysis of 2016 survey (14–15 year olds):						
Height‐for‐age Z‐score (HAZ)	18.6	4.7	0.162	0.011	0	0.890
BMI‐for‐age Z‐score (BMIZ)	18.6	4.7	0.162	0.011	0	0.889
PPVT percentage score (0–100)	18.6	4.7	0.162	0.010	0	0.915
Reading percentage score (0–100)	18.6	4.8	0.162	0.011	0	0.865
Mathematics percentage score (0–100)	18.6	4.7	0.162	0.011	0	0.888
Highest schooling grade attained	18.6	5	0.162	0.012	0	0.826

note: Bias is measured as the standardized percentage difference in the average value of the variable between the Hib‐vaccinated and ‐unvaccinated samples. Matching was based on propensity scores, using the kernel (Epanechnikov) matching method. Model (2) included public healthcare access indicators in propensity score estimation.

**Table 6:** Model (1)—standardized percentage bias before and after matching

	2013 Survey (11–12 year olds)		2016 Survey (14–15 year olds)	
	% bias before matching	% bias after matching	% bias before matching	% bias after matching
Age of child in months	−5.1	−2.4	−7.6	−0.7
Squared age of child in months	−5.1	−2.4	−7.6	−0.7
Whether child was born premature	10	1.1	10	4.5
Whether child is female	9.9	−1.8	9.3	−0.3
Rural household	−41.4	−5.3	−34.1	−2.6
Household size	−7	4	−14.8	2.6
Whether scheduled caste (SC)	−12.2	0.4	−12.2	−0.2
Whether scheduled tribe (ST)	13.3	−5.8	13.3	−4.2
Whether other backward classes (OBCs)	−4	2.2	−4	3.3
Whether Muslim	7.5	−0.8	7.5	−4.4
Whether Christian	−9	0.6	−9	0.6
Whether Buddhist	6.3	3.5	6.3	1.8
Whether household head is female	3.4	−2.6	8.7	−2.7
Age of household head in years	−4	2.5	−5.1	2.3
Head's schooling: <6 years	−13.8	−0.9	−12.7	−4.6
Head's schooling: ≥6 years but ≤11 years	−4.9	0.8	1.5	−0.7
Head's schooling: ≥12 years	19.7	3.5	20.3	7.3
Mother's schooling: <6 years	−4.1	−4.8	−5.9	−5.6
Mother's schooling: ≥6 years but ≤11 years	34.9	4.5	31.4	4.3
Mother's schooling: ≥12 years	−0.4	3.9	5.4	4.2
Mother's age in years	−11.8	2	−10.6	2.9
Household belongs to wealth quintile 2	−24.5	0.4	−12	−2.2
Household belongs to wealth quintile 3	1	−2.1	2.6	−2
Household belongs to wealth quintile 4	4.4	−1.6	1.9	−2.6
Household belongs to wealth quintile 5	32.9	4.6	33.8	5.6

note: Bias is measured as the standardized percentage difference in the average value of the variable between the Hib‐vaccinated and ‐unvaccinated samples. Results from the analysis of height‐for‐age Z score (HAZ) are shown. Matching was based on propensity scores, using the kernel (Epanechnikov) matching method. Model (1) excluded public healthcare access indicators from propensity score estimation.

**Table 7:** Model (2)—standardized percentage bias before and after matching

	2013 Survey (11–12 year olds)		2016 Survey (14–15 year olds)	
	% bias before matching	% bias after matching	% bias before matching	% bias after matching
Age of child in months	−6.1	−5.5	−6.3	−5.2
Squared age of child in months	−6.1	−5.5	−6.3	−5.1
Whether child was born premature	8.9	2	7.7	0.2
Whether child is female	10.7	3.1	11.3	4.7
Rural household	−52.7	−6.9	−47.4	−9.1
Household size	−7.3	1.4	−16.7	1.3
Whether scheduled caste (SC)	−11.7	−0.8	−14.2	−0.7
Whether scheduled tribe (ST)	16.4	−16.6	16.1	−16.5
Whether other backward classes (OBCs)	−9.4	9.7	−7.5	10.9
Whether Muslim	14.3	−2.2	16.7	−4.2
Whether Christian	−9.6	−3.5	−9.8	−5.7
Whether Buddhist	5.3	2.6	3.7	2.9
Whether household head is female	1.7	−2.7	10.1	−0.8
Age of household head in years	−6.5	6.3	−3	6
Head's schooling: <6 years	−14.3	−4.9	−11.2	−3.6
Head's schooling: ≥6 years but ≤11 years	−7.1	−0.5	3	−2.3
Head's schooling: ≥12 years	21.3	7.6	21	10.7
Mother's schooling: <6 years	−0.4	−2.6	−2.7	−3.4
Mother's schooling: ≥6 years but ≤11 years	33.2	1.7	30	2.6
Mother's schooling: ≥12 years	1.2	6.3	5.7	7.8
Mother's age in years	−10.6	3.1	−11	3.6
Household belongs to wealth quintile 2	−24.2	−1.4	71.5	4.7
Household belongs to wealth quintile 3	−30.8	0.5	64.9	3.5
Household belongs to wealth quintile 4	7.6	2.2	61.8	4.5
Household belongs to wealth quintile 5	47.8	8.3	−16.3	−1.7
Public hospital available in community	67.5	4	5.2	−1.7
Public health post available in community	62.4	2.7	4.3	5.8
Public health center available in community	60.5	3.8	34.6	2

note: Bias is measured as the standardized percentage difference in the average value of the variable between the Hib‐vaccinated and ‐unvaccinated samples. Results from the analysis of height‐for‐age Z score (HAZ) are shown. Matching was based on propensity scores, using the kernel (Epanechnikov) matching method. Model (2) included public healthcare access indicators in propensity score estimation.

Table 8 presents results from PSM using alternative matching algorithms. The associations of Hib vaccination in these additional analyses were similar in magnitude and statistical significance as in the original kernel PSM, with some minor variations.

**Table 8:** Alternative matching algorithms

	One‐to‐one neighbor matching				One‐to‐three neighbor matching			
	Model (1)		Model (2)		Model (1)		Model (2)	
	ATT	*P* value	ATT	*P* value	ATT	*P* value	ATT	*P* value
2013 survey (11–12 year olds):								
Height‐for‐age Z‐score (HAZ)	0.20	0.00	0.18	0.05	0.22	0.00	0.22	0.01
BMI‐for‐age Z‐score (BMIZ)	−0.04	0.66	−0.09	0.41	−0.02	0.80	−0.03	0.73
English percentage score (0–100)	3.48	0.01	1.49	0.25	3.71	0.00	3.79	0.00
PPVT percentage score (0–100)	−1.02	0.32	−0.65	0.57	−0.74	0.40	−0.72	0.47
Language percentage score (0–100)	1.07	0.35	−0.77	0.62	1.26	0.22	0.46	0.72
Mathematics percentage score (0–100)	5.82	0.00	2.33	0.16	4.90	0.00	3.03	0.04
Highest schooling grade attained	0.18	0.04	0.18	0.06	0.14	0.07	0.13	0.11
2016 survey (14–15 year olds):								
Height‐for‐age Z‐score (HAZ)	0.16	0.02	0.13	0.09	0.17	0.01	0.17	0.01
BMI‐for‐age Z‐score (BMIZ)	0.04	0.68	0.19	0.07	0.02	0.79	0.19	0.05
PPVT percentage score (0–100)	0.50	0.64	1.75	0.23	0.32	0.69	0.79	0.49
Reading percentage score (0–100)	4.20	0.00	3.62	0.03	4.01	0.00	2.46	0.05
Mathematics percentage score (0–100)	4.71	0.00	3.63	0.01	2.98	0.00	3.05	0.01
Highest schooling grade attained	0.22	0.05	0.14	0.37	0.16	0.03	0.14	0.05

note: Data are from 2013 and 2016 rounds of the Young Lives Survey, comprising 1824 children in the younger cohort in India. ATT denotes the propensity score matching estimator of the associations of Hib vaccination. One‐to‐one nearest neighbor matching with replacement and within a radius of 0.01 and one‐to‐three nearest neighbor matching were used. Model (1) excluded public healthcare access indicators from propensity score estimation, while Model (2) included them.

## Discussion and conclusion

Indian children who received the Hib vaccine in early childhood were found to be taller than similar Hib‐unvaccinated children at ages 11–12 and 14–15 years by 11–16%. Hib‐vaccinated children also scored 7–12% higher on English and mathematics tests at age 11–12 years and 7–10% higher on reading and mathematics tests at age 14–15 years and attained more schooling (by 11–12%) at both ages as compared with their unvaccinated counterparts. The estimates were smaller, and no statistically significant association of Hib vaccine receipt with schooling grade attainment was evident in the subsample of children with data on access to public healthcare facilities. Overall, our findings were not sensitive to the choice of matching algorithm and were statistically significant, even after accounting for possible confounding effects from BCG and measles vaccinations.

The burden of Hib disease in India was high in 2000—the only year during early life of the YLS cohort for which such data are available. Estimates from two modeling studies and the World Health Organization range from 2 to 4.5 million Hib cases in India in 2000, most of which were Hib pneumonia.^3^, ^24^, ^42^ Two‐thirds of Hib cases are known to occur in children under the age of 2 years, implying that a substantial proportion of the under‐2 cohort of 36.2 million Indian children at that time was affected.^43^ However, the burden of Hib meningitis may have been substantially underestimated in these models, as it was estimated from small surveillance studies which are known to capture such data inadequately.^24^ Hib nonpneumonia and nonmeningitis disease burden, which was calculated based on meningitis burden, was also likely underestimated.^24^

The positive association between Hib vaccination and anthropometric outcomes in our study could be mediated largely by pneumonia risk factors. In LMICs, stunted and underweight children are known to be at a higher risk of pneumonia.^44^, ^45^ The Hib‐unvaccinated children in our study were from lower‐income families and may have had higher rates of undernutrition before the 2006 survey, as compared with the Hib‐vaccinated children. As a result, they may have been predisposed to higher rates of pneumonia. With respect to cognitive test and schooling grade outcomes of children, the estimated positive associations of Hib vaccination could be mediated by worse nutritional status preschool and school absenteeism due to pneumonia among control group children.^12^, ^13^, ^14^

Our findings contribute substantially to the scant literature on the potential long‐term benefits of childhood vaccines in LMICs. In addition to the aforementioned study of Hib vaccination and anthropometric outcomes of Indian children aged 4–6,^25^ other studies have demonstrated commensurate benefits: measles vaccination was associated with 7.4% higher schooling enrollment among 8‐ to 16‐year‐old children in Bangladesh and 0.2 more schooling grades attained by 6‐ to 11‐year‐old children in South Africa,^46^, ^47^ and full immunization with 0.5 standard deviation higher cognitive test scores of 10‐ to 11‐year‐old Filipino children.^48^ In India, a study has linked the national immunization program with reduction in height‐for‐age deficit of under‐4 children by up to 25% and weight‐for‐age deficit by up to 15%.^49^

The findings have important policy implications. Worldwide, 191 countries had adopted the Hib vaccine by 2017, with an estimated 72% of Hib third dose (Hib3)—which promotes durability following the immune response built by the first two doses—coverage among 12‐ to 23‐month‐old children.^5^, ^50^ However, coverage varied widely, from 94% in high‐income countries to 81%, 77%, and 41%, respectively, in lower‐middle‐, low‐, and upper‐middle‐income countries,^50^ and 975,000 cases and 29,800 deaths from Hib still occurred among 1‐ to 59‐month‐old children worldwide in 2015.^3^ India's Hib3 coverage rates among children under the age of 2 years increased from 20% in 2013 to 88% in 2017,^5^ but its burden of disease remains the highest in the world.^3^ Funding for India's national immunization program remains inadequate. During 2013–2017, the estimated annual budgetary shortfall for the program increased from $8.6 to $544.2 million (in 2013 US$), primarily because of the introduction of pentavalent and other new vaccines.^51^, ^52^

The economic viability of publicly funded vaccines is traditionally evaluated using cost‐effectiveness analysis (CEA). However, CEAs that use disability‐adjusted life years (DALYs) to measure health gains have many methodological shortcomings, chief among them being that DALYs do not account for potential nonhealth benefits of vaccines and ignore any equity aspects of benefits across population subgroups. Therefore, alternative methodologies, such as benefit–cost analysis or multicriteria decision analysis, which incorporate various health and nonhealth metrics, have gained prominence in recent years.^53^, ^54^, ^55^ Our findings show that the Hib vaccine could rank highly in the context of multicriteria‐based resource allocation.

Our analysis has some limitations. Although we included an extensive set of child, household, and healthcare access covariates in our PSM, unobserved heterogeneity may be present that could bias the results. For example, parents may have vaccinated children based on their health (i.e., perceived likelihood of survival, susceptibility to infections, and growth), which was unobserved in the data. Also, if Hib vaccination of a child was associated positively with other human capital investments that also affect long‐term outcomes (e.g., nutrition or healthcare resources), our estimates may be inflated. PSM would not mitigate any possible biases arising from such unobserved differences between Hib‐vaccinated and Hib‐unvaccinated children.

Whether the reported receipt of Hib vaccine in YLS was cross‐validated with vaccination cards is unknown, making it susceptible to measurement errors. Some Hib‐unvaccinated children may have been categorized as vaccinated, making our estimates of positive associations conservative. The survey also did not collect information on age at vaccination or the number of Hib doses administered to children. The 2006 YLS asked about the receipt of Hib vaccine (without specifying dose) in the 4 years preceding the interview date; considering that the children were 4–6 years old in 2006, the survey may have missed Hib vaccinations during the first 2 years of life of some children. Because these children would have been improperly categorized as Hib‐unvaccinated, our estimates of the positive associations of the vaccine may be conservative.

Large regional disparities exist in India with respect to socioeconomic indicators, access to and quality of healthcare and schooling infrastructure, and disease burden. For example, children from the northern Indian states, such as Uttar Pradesh, Bihar, and Madhya Pradesh, have higher rates of undernutrition and incidence of childhood diseases. Therefore, our findings from Andhra Pradesh may not be generalizable to other parts of India.

Further, owing to a lack of data, we could not capture any secondary immunity provided by the Hib vaccine to Hib‐unvaccinated children who might have been in contact with Hib‐vaccinated children. The 2013 and 2016 YLS surveys also could not capture data on children who had died since 2006.^28^ These may have made our estimates of the associations of the vaccine conservative.

Notwithstanding these limitations, our findings do suggest that Hib vaccination not only reduces the immediate burden of Hib diseases, but may also be associated with improved adolescent height, cognitive functioning, and schooling attainment in India. These positive health and social outcomes are strongly associated with positive economic outcomes, implying that the benefits of Hib vaccination for LMICs could reverberate throughout multiple domains of society. India has made great strides in its struggle against Hib, but our analysis suggests the need to continue, indeed accelerate, efforts to expand Hib vaccination.

## Competing interests

D.B. has previously received research support or personal fees from GlaxoSmithKline plc, Merck, Pfizer, and Sanofi‐Pasteur related generally to value‐of‐vaccination research, but not for this study. All other authors declare no competing interests.

## References

[1] CDC . 2018 Vaccine information statement—*Haemophilus influenzae* type b. Accessed December 19, 2018. https://www.cdc.gov/vaccines/hcp/vis/vis-statements/hib.html.

[2] Knoll, M.D. , K.L. O'Brien , E. Henkle , *et al* 2009 Global Literature Review of Haemophilus influenzae Type b and Streptococcus pneumoniae Invasive Disease among Children Less than Five Years of Age 1980–2005. Geneva: World Health Organization.

[3] Wahl, B. , K.L. O'Brien , A. Greenbaum , *et al* 2018 Burden of *Streptococcus pneumoniae* and *Haemophilus influenzae* type b disease in children in the era of conjugate vaccines: global, regional, and national estimates for 2000–15. Lancet Glob. Health 6: e744–e757. 29903376 10.1016/S2214-109X(18)30247-X PMC6005122

[4] Kahn, G.D. , D. Thacker , S. Nimbalkar & M. Santosham . 2014 High cost is the primary barrier reported by physicians who prescribe vaccines not included in India's Universal Immunization Program. J. Trop. Pediatr. 60: 287–291. 24567310 10.1093/tropej/fmu012

[5] WHO‐UNICEF . 2018 WHO immunization coverage estimates. World Health Organization. Accessed October 26, 2017. https://www.who.int/immunization/monitoring_surveillance/routine/coverage/en/.

[6] Currie, J. & T. Vogl . 2013 Early‐life health and adult circumstance in developing countries. Annu. Rev. Econ. 5: 1–36.

[7] Almond, D. & J. Currie . 2011 Killing me softly: the fetal origins hypothesis. J. Econ. Perspect. 25: 153–172. 25152565 10.1257/jep.25.3.153 PMC4140221

[8] Dewey, K.G. & K. Begum . 2011 Long‐term consequences of stunting in early life. Matern. Child Nutr. 7(Suppl. 3): 5–18. 21929633 10.1111/j.1740-8709.2011.00349.x PMC6860846

[9] Grimwood, K. & A.B. Chang . 2015 Long‐term effects of pneumonia in young children. Pneumonia 6: 101. 31641584 10.15172/pneu.2015.6/671 PMC5922344

[10] Edmond, K. , S. Scott , V. Korczak , *et al* 2012 Long term sequelae from childhood pneumonia; systematic review and meta‐analysis. PLoS One 7: e31239. 22384005 10.1371/journal.pone.0031239 PMC3285155

[11] Schlaudecker, E.P. , M.C. Steinhoff & S.R. Moore . 2011 Interactions of diarrhea, pneumonia, and malnutrition in childhood: recent evidence from developing countries. Curr. Opin. Infect. Dis. 24: 496–502. 21734569 10.1097/QCO.0b013e328349287d PMC5454480

[12] Saliba, G.S. , W.P. Glezen & T.D.Y. Chin . 1967 Etiologic studies of acute respiratory illness among children attending public schools. Am. Rev. Respir. Dis. 95: 592–602. 6021120 10.1164/arrd.1967.95.4.592

[13] Farha, T. & A.H. Thomson . 2005 The burden of pneumonia in children in the developed world. Paediatr. Respir. Rev. 6: 76–82. 15911451 10.1016/j.prrv.2005.03.001

[14] Bhalotra, S.R. & A. Venkataramani . 2011 The Captain of the Men of Death and His Shadow: Long‐Run Impacts of Early Life Pneumonia Exposure. Rochester, NY: Social Science Research Network.

[15] D'Angio, C.T. , R.G. Froehlke , G.A. Plank , *et al* 1995 Long‐term outcome of *Haemophilus influenzae* meningitis in Navajo Indian children. Arch. Pediatr. Adolesc. Med. 149: 1001–1008. 7655584 10.1001/archpedi.1995.02170220067009

[16] Bedford, H. , J. de Louvois , S. Halket , *et al* 2001 Meningitis in infancy in England and Wales: follow up at age 5 years. BMJ 323: 533. 11546697 10.1136/bmj.323.7312.533 PMC48156

[17] Koomen, I. , D.E. Grobbee , J.J. Roord , *et al* 2004 Prediction of academic and behavioural limitations in school‐age survivors of bacterial meningitis. Acta Paediatr. 93: 1378–1385. 15499961

[18] Taylor, H.G. , C. Schatschneider , S. Petrill , *et al* 1996 Executive dysfunction in children with early brain disease: outcomes post *Haemophilus influenzae* meningitis. Dev. Neuropsychol. 12: 35–51.

[19] Grimwood, K. , P. Anderson , V. Anderson , *et al* 2000 Twelve year outcomes following bacterial meningitis: further evidence for persisting effects. Arch. Dis. Child. 83: 111–116. 10906014 10.1136/adc.83.2.111 PMC1718445

[20] Petersen, H. , M. Patel , E.F. Ingason , *et al* 2014 Long‐term effects from bacterial meningitis in childhood and adolescence on postural control. PLoS One 9: e112016. 25405756 10.1371/journal.pone.0112016 PMC4236047

[21] Anderson, V. , P. Anderson , K. Grimwood & T. Nolan . 2004 Cognitive and executive function 12 years after childhood bacterial meningitis: effect of acute neurologic complications and age of onset. J. Pediatr. Psychol. 29: 67–81. 15096529 10.1093/jpepsy/jsh011

[22] Bijlmer, H.A. , L. van Alphen , B.M. Greenwood , *et al* 1990 The epidemiology of *Haemophilus influenzae* meningitis in children under five years of age in The Gambia, West Africa. J. Infect. Dis. 161: 1210–1215. 2345302 10.1093/infdis/161.6.1210

[23] Mwangi, I. , J. Berkley , B. Lowe , *et al* 2002 Acute bacterial meningitis in children admitted to a rural Kenyan hospital: increasing antibiotic resistance and outcome. Pediatr. Infect. Dis. J. 21: 1042–1048. 12442027 10.1097/00006454-200211000-00013

[24] Watt, J.P. , L.J. Wolfson , K.L. O'Brien , *et al* 2009 Burden of disease caused by *Haemophilus influenzae* type b in children younger than 5 years: global estimates. Lancet 374: 903–911. 19748399 10.1016/S0140-6736(09)61203-4

[25] Upadhyay, A.K. & S. Srivastava . 2017 Association between *Haemophilus influenza* type B (Hib) vaccination and child anthropometric outcomes in Andhra Pradesh (India): evidence from the Young Lives Study. J. Public Health 25: 581–589.

[26] Boyden, J. 2016 Young Lives: an international study of childhood poverty: rounds 1–4 constructed files, 2002–2014 [data collection]. 2nd ed UK Data Service.

[27] Barnett, I. , P. Ariana , S. Petrou , *et al* 2013 Cohort profile: the Young Lives Study. Int. J. Epidemiol. 42: 701–708. 22617687 10.1093/ije/dys082

[28] Galab, S. , P. Prudhvikar Reddy & R. Singh . 2014 Young Lives Survey design and sampling in Andhra Pradesh, India. Young Lives, Oxford Department of International Development (ODID), University of Oxford.

[29] NFHS . 2000 National Family Health Survey (NFHS‐2), 1998–99: India. International Institute for Population Sciences (IIPS) and ORC Macro. Mumbai: IIPS.

[30] Cueto, S. & J. León . 2012 Psychometric characteristics of cognitive development and achievement instruments in round 3 of Young Lives. Young Lives. Accessed April 27, 2018. https://www.younglives.org.uk/sites/www.younglives.org.uk/files/YL-TN25_Cueto.pdf.

[31] Crookston, B.T. , W. Schott , S. Cueto , *et al* 2013 Postinfancy growth, schooling, and cognitive achievement: young Lives. Am. J. Clin. Nutr. 98: 1555–1563. 24067665 10.3945/ajcn.113.067561 PMC3831540

[32] Sharma, A. , W.A. Kaplan , M. Chokshi , *et al* 2015 Implications of private sector Hib vaccine coverage for the introduction of public sector Hib‐containing pentavalent vaccine in India: evidence from retrospective time series data. BMJ Open 5: e007038. 10.1136/bmjopen-2014-007038 PMC4342586 25712822

[33] Laxminarayan, R. & N.K. Ganguly . 2011 India's vaccine deficit: why more than half of Indian children are not fully immunized, and what can—and should—be done. Health Aff. 6: 1096–1103. 10.1377/hlthaff.2011.0405 21653963

[34] Singh, P.K. 2013 Trends in child immunization across geographical regions in India: focus on urban–rural and gender differentials. PLoS One 8: e73102. 24023816 10.1371/journal.pone.0073102 PMC3762848

[35] Vikram, K. , R. Vanneman & S. Desai . 2012 Linkages between maternal education and childhood immunization in India. Soc. Sci. Med. 75: 331–339. 22531572 10.1016/j.socscimed.2012.02.043 PMC3495071

[36] Khan, J. , A. Shil & R. Prakash . 2018 Exploring the spatial heterogeneity in different doses of vaccination coverage in India. PLoS One 13: e0207209. 30485291 10.1371/journal.pone.0207209 PMC6261550

[37] Dehejia, R.H. & S. Wahba . 2002 Propensity score‐matching methods for nonexperimental causal studies. Rev. Econ. Stat. 84: 151–161.

[38] Dehejia, R.H. & S. Wahba . 1999 Causal effects in nonexperimental studies: reevaluating the evaluation of training programs. J. Am. Statist. Assoc. 94: 1053–1062.

[39] Heckman, J.J. , H. Ichimura & P.E. Todd . 1997 Matching as an econometric evaluation estimator: evidence from evaluating a job training programme. Rev. Econ. Stud. 64: 605–654.

[40] Rosenbaum, P.R. & D.B. Rubin . 1983 The central role of the propensity score in observational studies for causal effects. Biometrika 70: 41–55.

[41] Briones, K. 2017 How many rooms are there in your house? Constructing the Young Lives Wealth Index. Young Lives, Oxford Department of International Development (ODID), University of Oxford.

[42] WHO . 2018 Estimated Hib and pneumococcal deaths for children under 5 years of age, 2000. World Health Organization. Accessed March 17, 2019. https://www.who.int/immunization/monitoring_surveillance/burden/estimates/Pneumo_hib_2000/en/.

[43] Census of India . 2001 Population in single year age by residence and sex, 2001—INDIA. Government of India. Accessed May 16, 2019. https://data.gov.in/resources/population-single-year-age-residence-and-sex-2001-india.

[44] Victora, C.G. , B.R. Kirkwood , A. Ashworth , *et al* 1999 Potential interventions for the prevention of childhood pneumonia in developing countries: improving nutrition. Am. J. Clin. Nutr. 70: 309–320. 10479192 10.1093/ajcn/70.3.309

[45] Kirkwood, B.R. , S. Gove , S. Rogers , *et al* 1995 Potential interventions for the prevention of childhood pneumonia in developing countries: a systematic review. Bull. World Health Organ. 73: 793–798. 8907773 PMC2486683

[46] Anekwe, T.D. , M.‐L. Newell , F. Tanser , *et al* 2015 The causal effect of childhood measles vaccination on educational attainment: a mother fixed‐effects study in rural South Africa. Vaccine 33: 5020–5026. 25936663 10.1016/j.vaccine.2015.04.072 PMC4570928

[47] Driessen, J. , A. Razzaque , D. Walker & D. Canning . 2015 The effect of childhood measles vaccination on school enrolment in Matlab, Bangladesh. Appl. Econ. 47: 6019–6040.

[48] Bloom, D.E. , D. Canning & E.S. Shenoy . 2012 The effect of vaccination on children's physical and cognitive development in the Philippines. Appl. Econ. 44: 2777–2783.

[49] Anekwe, T.D. & S. Kumar . 2012 The effect of a vaccination program on child anthropometry: evidence from India's Universal Immunization Program. J. Public Health (Oxf.) 34: 489–497. 22611261 10.1093/pubmed/fds032

[50] WHO . 2018 Immunization coverage—key facts, 16 July 2018. World Health Organization. Accessed December 27, 2018. https://www.who.int/news-room/fact-sheets/detail/immunization-coverage.

[51] Chatterjee, S. , M. Pant , P. Haldar , *et al* 2016 Current costs & projected financial needs of India's Universal Immunization Programme. Indian J. Med. Res. 143: 801–808. 27748306 10.4103/0971-5916.192073 PMC5094121

[52] Chatterjee, S. , P. Das , A. Nigam , *et al* 2018 Variation in cost and performance of routine immunisation service delivery in India. BMJ Glob. Health 3: e000794. 10.1136/bmjgh-2018-000794 PMC6014207 29946488

[53] Jehu‐Appiah, C. , R. Baltussen , C. Acquah , *et al* 2008 Balancing equity and efficiency in health priorities in Ghana: the use of multicriteria decision analysis. Value Health 11: 1081–1087. 19602214 10.1111/j.1524-4733.2008.00392.x

[54] Baltussen, R. , A.H.A. ten Asbroek , X. Koolman , *et al* 2007 Priority setting using multiple criteria: should a lung health programme be implemented in Nepal? Health Policy Plan 22: 178–185. 17412742 10.1093/heapol/czm010

[55] Verguet, S. , R. Laxminarayan & D.T. Jamison . 2014 Universal public finance of tuberculosis treatment in India: an extended cost‐effectiveness analysis. Health Econ. 24: 318–332. 24497185 10.1002/hec.3019

